# Automatic Training of Rat Cyborgs for Navigation

**DOI:** 10.1155/2016/6459251

**Published:** 2016-06-29

**Authors:** Yipeng Yu, Zhaohui Wu, Kedi Xu, Yongyue Gong, Nenggan Zheng, Xiaoxiang Zheng, Gang Pan

**Affiliations:** ^1^College of Computer Science and Technology, Zhejiang University, Hangzhou 310027, China; ^2^Qiushi Academy for Advanced Studies, Zhejiang University, Hangzhou 310027, China

## Abstract

A rat cyborg system refers to a biological rat implanted with microelectrodes in its brain, via which the outer electrical stimuli can be delivered into the brain in vivo to control its behaviors. Rat cyborgs have various applications in emergency, such as search and rescue in disasters. Prior to a rat cyborg becoming controllable, a lot of effort is required to train it to adapt to the electrical stimuli. In this paper, we build a vision-based automatic training system for rat cyborgs to replace the time-consuming manual training procedure. A hierarchical framework is proposed to facilitate the colearning between rats and machines. In the framework, the behavioral states of a rat cyborg are visually sensed by a camera, a parameterized state machine is employed to model the training action transitions triggered by rat's behavioral states, and an adaptive adjustment policy is developed to adaptively adjust the stimulation intensity. The experimental results of three rat cyborgs prove the effectiveness of our system. To the best of our knowledge, this study is the first to tackle automatic training of animal cyborgs.

## 1. Introduction

With advances in brain-machine interfaces (BMIs), neural engineering, and artificial intelligence, biorobots are becoming more and more attractive. A biorobot uses an animal as a host and controls the host via neural interfaces. Biorobots are superior in many aspects to traditional mechanical robots, such as mobility, perceptivity, adaptability, and energy consumption. In the recent two decades, biorobots have been implemented on different kinds of creatures, such as cockroaches [1], moths [2, 3], sharks [4], rats [5–8], geckos [9], and beetles [10].

Rat cyborg is one of typical biorobots [5]. It has shown a great potential in various applications, including search and rescue in disaster areas, geographic information collection, explosives detection, and landmine detection [11, 12]. Nowadays researchers primarily use three navigation commands (FORWARD, LEFT, and RIGHT) to steer a rat cyborg [5, 7, 13–15], pioneered by Talwar et al. [5]. Lin et al. developed the STOP navigation command by electrical stimulation in the dorsolateral periaqueductal gray (dlPAG) of the brain [16]. Although the optical neural control technology [17] has been explored for rat cyborg control, electrical stimulation is currently the primary way to control a rat cyborg.

Before a rat cyborg can be used for navigation, a manual training process is needed to reinforce the desired behaviors (turning left, turning right, and moving forward) by pairing the behaviors with the corresponding electrical stimuli (LEFT, RIGHT, and FORWARD). Navigation training is necessary not only for new rat cyborgs, but also for trained rat cyborgs. A new untrained rat cyborg needs to go through an entire training procedure to establish connections between the behaviors and the electrical stimuli, which often takes 1-2 weeks, with 1-2 hours per day. A trained rat cyborg also needs retraining to keep the connections.

During the manual training, the trainer has to keep watching the rat cyborg and send the control commands of electrical stimuli repeatedly. There are three major problems of manual training. First, the trainer should be professional in rat cyborg training. It is hard for an inexperienced person to train a rat cyborg well. Second, the trainer is required to be highly concentrated all the time. The fatigue may lead to some manual misoperation, since the whole procedure is very time-consuming and tedious. Third, the learning states and behaviors of the rat cyborg cannot be recorded for quantitative analysis and personalized parameter configuration, which may be very helpful for further research.

To address these problems, we develop a vision-based automatic training system, which aims to free the trainers and present a better training result compared to manual training. In the system, the behaviors of a rat cyborg are monitored by a camera and analyzed by a computer in real time. Then, based on the analyzed states, the computer will continuously make decisions to generate training tasks for the rat cyborg. A hierarchical training framework which has a reactive layer and a deliberative layer is introduced. Based on the framework, our training system is able to output real-time electrical stimulation to train rat cyborgs. The experimental results on navigation show that our method successfully trained the rat cyborgs in a short time. In addition, the behavior changes and learning curves recorded during the training procedure are also discussed.

## 2. An Overview of Manual Navigation Training

### 2.1. Principles of Navigation Training

**Figure 1:**
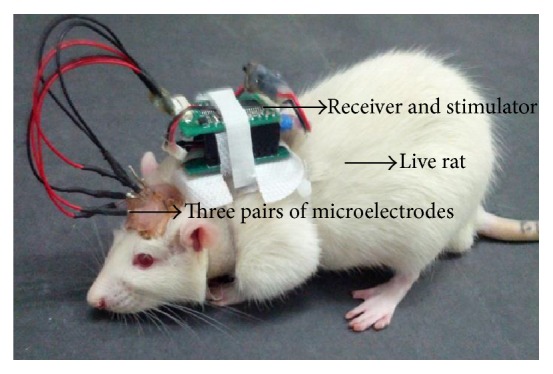
Rat cyborg.

Our rat cyborgs were developed based on the previous work in [7]. An illustration of the rat cyborg is shown in Figure 1. A pair of microstimulating electrodes were implanted into the medial forebrain bundle (MFB) of the rat's brain. The other two pairs were implanted into the whisker barrel fields of left and right somatosensory cortices (SI). After 5 days of recovery, a wireless microstimulator was mounted on the back of the rat to deliver electrical stimuli into the brain via the implanted electrodes. This allowed the user, using a computer, to deliver the stimulus pulses (pulse interval: 10 ms, pulse duration: 1 ms, pulse number: 10–15, and pulse amplitude: 1–10 V, per train) to any of the implanted brain sites from distances of up to 100 m away via Bluetooth. The intensity of stimulus pulses is determined by the pulse number and pulse amplitude. Stimulation in MFB can excite the rat cyborg by increasing the level of dopamine in its brain, and stimulation in the left or right SI makes the rat cyborg feel as if its whiskers were touching a barrier in the corresponding side [18, 19].

Operant conditioning is often used to train a desired behavior in an animal [20]. In the rat cyborg navigation training, MFB stimulation is used as the reward as well as a cue (FORWARD) to move ahead [21–23]. Left and right SI stimulation are used as the cues (LEFT and RIGHT) to turn left and turn right. In order to get the reward, the rat cyborg needs to learn to do the correct behaviors corresponding to the cues. The behavior of moving ahead is trained by “FORWARD-moving ahead-reward” procedure, the behavior of turning left is trained by “LEFT-turning left-reward” procedure, and the behavior of turning right is trained by “RIGHT-turning right-reward” procedure. After sufficient behavior training, a rat cyborg would turn left (right) in response to the LEFT (RIGHT) cue and move ahead in response to the FORWARD cue. A well-trained rat cyborg can follow the remote brain stimulation as instructions to direct its movements. During the training procedure, the intensity of LEFT or RIGHT remains unchanged for SI stimulation just acts as a cue, but the intensity (determined by the pulse number and pulse amplitude) of the reward stimulation (FORWARD) should be increased (to activate the rat cyborg) or decreased (to inactivate the rat cyborg) according to the learning states of the rat cyborg.

### 2.2. Training Procedures

**Figure 2:**
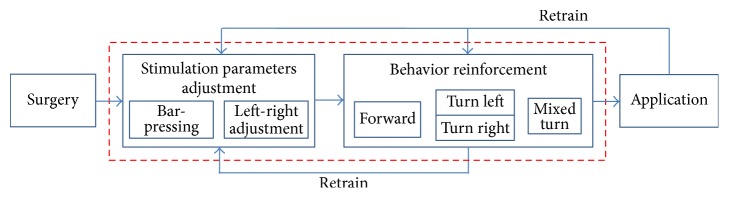
Manual training procedure.

The manual training procedure is illustrated in Figure 2. The microstimulating electrodes were implanted into the brain in surgery procedure. The retrain arrows indicate that a trained rat cyborg needs retraining. The two procedures enclosed in the rectangle in red dot are the most important parts and will be described in detail below.

The stimulation parameters adjustment procedure is to heuristically find a set of optimal stimulation parameters for a rat cyborg. It consists of bar-pressing and left-right adjustment. If a stimulation is too mild, it will be inadequate to excite the rat cyborg; otherwise, if it is too strong, the rat cyborg will be injured. The optimal intensity of the reward stimulation was figured out in the bar-pressing procedure, and the optimal intensity of the somatosensory cue stimulation was figured out in the left-right adjustment procedure.

Behavior reinforcement procedure is to teach the rat cyborg to make the correct response to the corresponding stimulation. The training environment used in this procedure is an eight-arm radial maze, which has been used in many behavior studies and can be seen as a consecutively joined structure of eight T mazes. There are three tasks in this procedure:

(i) *Forward (T0).* A task named T0 was to train the behavior of moving ahead. With the individual cue of FORWARD, the rat cyborg should go through all the eight arms of the maze one by one clockwise or anticlockwise without any incorrect turns.
(ii) *Turn Left and Turn Right (T1).* A task named T1 was to train the behaviors of turning left and turning right, respectively. With reward and LEFT, the rat cyborg should go through the eight arms one by one clockwise without any incorrect turns; with reward and RIGHT, the rat cyborg should go through the eight arms one by one anticlockwise without any incorrect turns. Concretely speaking, when the rat cyborg is going to leave the current maze arm (namely, in the front end of the maze arm), the LEFT or RIGHT cue will be sent to instruct it. If the rat cyborg turns to the correct arm, the FORWARD stimulation will be sent to reward it. The individual reward stimulation will induce the rat cyborg to move to the tail end of the arm first and then to the front end of the arm again. On the other hand, if the rat cyborg turns to a wrong arm, it will not get any reward. When the rat cyborg turns around in the wrong arm, the individual FORWARD stimulation will be sent to induce the rat cyborg to return to the front end of the arm. Due to the surgery impact, before there is any training, under the reward stimulation, some rat cyborgs prefer to turn left while others prefer the opposite. If a rat cyborg preferred to turn left, in T1 we trained it to turn left first; otherwise right-turning training goes first. The first subtraining task in T1 was labeled as T1_*a*_, and the second was labeled as T1_*b*_.
(iii) *Mixed Turn (T2).* A task named T2 was to reinforce the behaviors of turning left and turning right simultaneously. With reward and random cues, the rat cyborg should turn to the left (right) arm under LEFT (RIGHT) stimulation without making any mistakes in 8 consecutive turns.

During the behavior reinforcement procedure, the trainer might adjust the intensity of the reward stimulation based on his own experience in rat cyborg navigation training. Upon completion of the T2 task, the rat cyborg will be ready for navigation.

## 3. Automatic Navigation Training

Manual training of a rat cyborg needs a lot of effort. We attempt to automate it with a computing system, replacing the manually time-consuming training procedure. Our basic idea is to keep the rat cyborg under surveillance via a camera and being trained by a computer in real time. The live rat cyborgs have self-consciousness, and they might underreact or overreact in the training procedure. Meanwhile, the body of a rat cyborg is nonrigid and it has various postures. Therefore, assigning the automatic training to a computer faces four major issues: (1) how to set training tasks in order to ensure that a rat cyborg is qualified for navigation; (2) how to sense motion and behaviors of the rat cyborg in real time; (3) how to detect abnormal learning states (underreaction or overreaction) of the rat cyborg in the training procedure; (4) and developing a smart stimulation strategy which is able to provide adaptive electrical stimuli. In this section, we first reset the training tasks in automatic training to settle the first issue and then propose an automatic training framework to settle the other three issues.

### 3.1. Training Tasks

**Figure 3:**
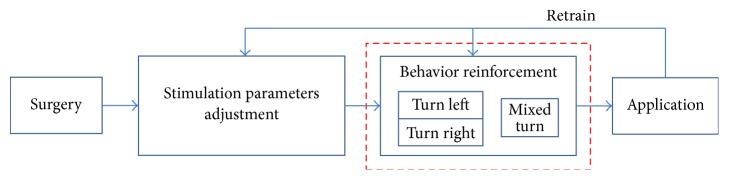
Automatic training procedure.

In our previous work on automatic training [24], we found that training of moving ahead behavior in the bar-pressing procedure and forward procedure (T0) was redundant, for this behavior was also trained in T1 and T2. We eliminated these two procedures, so different to the manual training, the training tasks in our automatic training consisted of only T1 and T2. In addition, without training in T1 first, we had failed to train two rat cyborgs directly with T2 in the previous work. It suggests that the process of rat cyborg training should follow in order and advance step by step. The whole procedure of automatic training is illustrated in Figure 3. The stimulation parameters adjustment procedure was to get the optimal intensity of the reward stimulation and somatosensory cue stimulation by manual test. The behavior reinforcement procedure, which took most of the training time (1-2 weeks) in the manual training, was automated in this study.

### 3.2. The Framework

**Figure 4:**
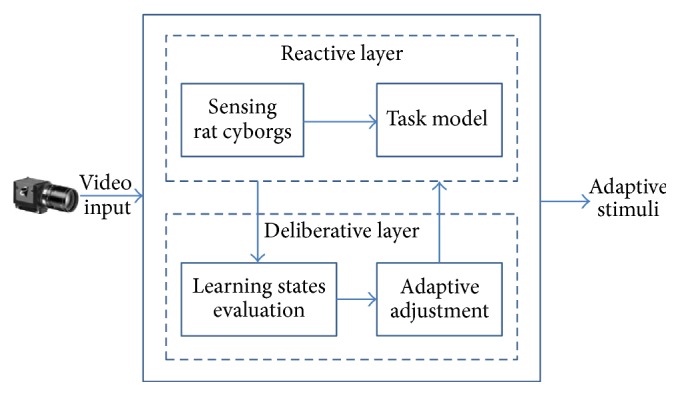
Automatic rat cyborg training framework.

A hierarchical automatic training framework is shown in Figure 4. The input of the framework is the images captured by a bird-eye camera mounted above the rat cyborg; the output is the adaptive stimuli. This framework has two layers. The reactive layer provides a real-time training guidance to the rat cyborg based on the sensing module and task model. It decides which stimulus (LEFT, RIGHT, or FORWARD) to send and when to stimulate the rat cyborg. The deliberative layer is responsible for adaptive adjustment of the reward stimulation according to the learning states of the rat cyborg. Each module of this framework is described in detail below.

#### 3.2.1. Sensing Rat Cyborgs

There are two main traditional methods for rat tracking. One is marking the rat head and rat body with a bright color [11]; the other one is placing pressure sensors under the rat foot. However, the color markers are easily to be covered by the rat body in the former method, while it is hard for the latter method to get accurate head position and body position of the rat. Replacing these two defective tracking methods, we developed new methods based on optical flow to get motion parameters (head position, body position, and heading direction) and behaviors of the rat. These methods were implemented with OpenCV library [25].

 (*1) Sensing Motion*. First of all, we need to know where the rat cyborg is (body position and head position) and to which direction it is heading in the training environment. The details of our motion-sensing algorithm are as follows.

(i) *Body Position.* After background subtraction, a small rectangle which is big enough to cover a rat cyborg is used to search the entire image, and the rectangle (R1) which has the most target pixels is saved. The mean position of the target pixels in R1 is calculated as the body position (P_*b*_).
(ii) *Head Position.* When tracking a moving target, feature points like corners are usually used [26]. Because the rat cyborg has a backpack, most of the corners appear around the backpack area which is very close to the head. Thus we can take the mean position of these corners as the head position. We adopted Shi-Tomasi feature detection algorithm so that good corners resulted as long as the smaller of the two eigenvalues was greater than a minimum threshold [27]. After we get the valid corners of a rat cyborg in R1, a smaller rectangle which is half of R1 is used to search the entire area of R1, and the rectangle (R2) which has the most corners is saved. The mean position of the corners in R2 is calculated as the head position (P_*h*_).
(iii) *Heading Direction.* The direction from P_*b*_ to P_*h*_ is taken as the heading direction of the rat cyborg (Θ_*r*_).

**Figure 5:**
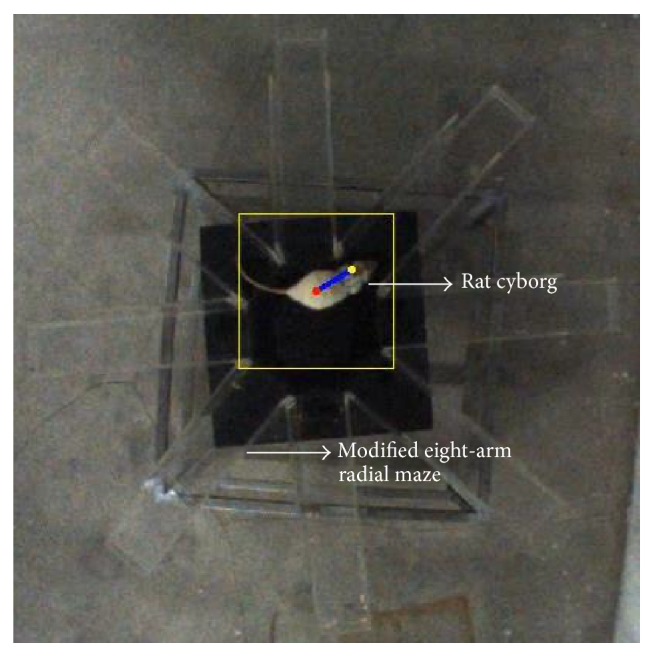
Motion parameters calculated by our method. The red point is the body position (P_*b*_), the yellow point is the head position (P_*h*_), and the blue line is the heading direction of the rat cyborg (Θ_*r*_).

Figure 5 is a demonstration of our tracking method. The position of the yellow rectangle would be updated according to the body location of the rat cyborg. This method has a tracking accuracy of 89.26%, as measured from ground-truth manual annotations of ten thousands video frames of the automatic training procedure. The correct tracking is defined as follows: P_*b*_ locates the body part of the rat cyborg, P_*h*_ locates the head part of the rat cyborg, and the deviation of Θ_*r*_ is no greater than 30 degrees. Most of the wrong tracking occurred at the tail end of the maze arms where the rat cyborg bent its body. The computation time between two frames varies from 15 ms to 47 ms, and the average computation time is 26.21 ms. The video frame rate in our system is 30 frames per second, leaving the time interval between frames equal to 33.33 ms. Thereby our rat cyborg tracking approach fits the speed of image acquisition.

*(2) Sensing Behaviors.* After we get the basic motion parameters, we also need to know what the rat cyborg is doing (i.e., behaviors). Rat cyborgs have various behaviors in the training environment, such as immobility, grooming, and climbing up the plexiglass wall [28]. In the manual training procedure, when a rat cyborg is in immobility, only the reward should be sent to excite the rat cyborg; when a rat cyborg is grooming or climbing, no stimuli should be sent. However, we did not automatically recognize grooming and climbing in our automatic training system (these two behaviors were counted manually for behavioral analysis). The reasons are as follows: first, real-time and accurate recognition of grooming and climbing in our training system is difficult for there is only one low-resolution camera mounted above the modified eight-arm radial maze; second, both grooming and climbing are short-time behaviors, so the failure to detect them should not have an impact on the automatic training.

A rat cyborg in immobility must be detected for it would hold still for a long time if it did not get any reward stimulation. Instead of using the locomotion speed of the body position to recognize the behavior of immobility, a more effective method was proposed in this study. Corners which were “easy to track” were calculated by Shi-Tomasi feature detection algorithm and among them we implemented Pyramid Lucas-Kanade's optical flow algorithm to get the number of the moving corners (*N*_*m*_) in the next frame [25]. The immobility behavior is detected if *N*_*m*_ is always equal to zero in the 100 consecutive frames (about 3.33 s). Performance of immobility recognition was tested using 700 video clips (about 40 minutes). Our method achieves a precision of 82.0%, a recall of 97.6%, and an accuracy of 97.1%. Detailed results are shown in Table 1.

#### 3.2.2. Task Model

**Figure 6:**
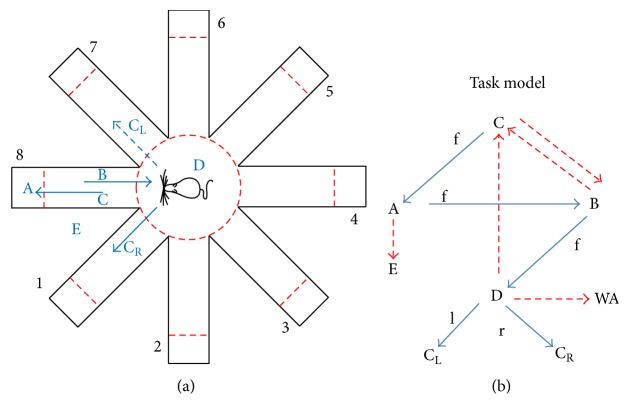
(a) An overhead view of the modified eight-arm radial maze and state division. A: at the tail end of the maze arms. B: in the passage of the arm and going inside. C: in the passage of the arm and going outside. C_L_: in the center circle and the current task is to turn left. C_R_: in the center circle and the current task is to turn right. D: in the center circle of the maze. E: out of the maze. (b) Task model. WA: other 5 wrong arms, f: FORWARD, l: LEFT, and r: RIGHT.

Task model decides which stimulus to send and when to stimulate the rat cyborg. The task environment of the automatic training is a modified eight-arm radial maze (see Figure 6(a)). Therefore the training tasks can be divided into eight subtasks. Taking training the behavior of turning right as example, it can be divided into eight subtasks: arms 1→2, 2→3, 3→4, 4→5, 5→6, 6→7, 7→8, and 8→1. Each subtask can be further divided into the transitions of five primary training states. Taking subtask 8→1 as example, it can be divided into D→C, C→A, A→B, B→D, and D→C_R_. The training states depend on the body position, head position, and heading direction of the rat cyborg, and their transitions depend on the current training tasks.

Rat cyborgs have their self-consciousness to move. They might move to another state without receiving any stimulus or move to a wrong state even with the correct stimulus. Thus training state transitions of our automatic training can be modeled as the task model shown in Figure 6(b). The state transitions in blue solid arrows are what we expect to see in the training procedure. With the hint of the direction stimulus (FORWARD, LEFT, or RIGHT), a rat cyborg that moves in accordance with the transitions indicated by the blue solid arrows would always get reward stimulation, but one that moves in accordance with the transitions indicated by the red-dotted arrows would not get any reward stimulation. A parameterized state machine which bases its state transitions on sensor readings and heuristics was chosen, since the structure of it is similar to the manual training and the expertise can be incorporated easily [29]. It can be easily implemented with a series of if/else rules. A number of parameters of the state machine are shown in Table S1 of the Supplementary Material available online at http://dx.doi.org/10.1155/2016/6459251. Default values of these parameters were set based on observation from manual training trials, discussions with expert trainers, and pre-experiment in our previous work [24].

#### 3.2.3. Learning States Evaluation

The task model provides a real-time training guidance to the training rat cyborg when it is in normal locomotion. When the rat cyborg is demonstrating some abnormal training behaviors, the learning states evaluation module will come into play. This module detects the abnormal learning states (underreaction or overreaction) of the rat cyborg based on the behavior of immobility and the current locomotion speed. In the training procedure, the rat cyborg tends to hold still if it had been familiar with the training environment. As mentioned before, if the rat cyborg is in immobility, the reward stimulation should be sent to excite the rat cyborg. Moreover, if the immobility behavior is too frequent in a trial, which means the rat cyborg is in underreaction, the intensity of reward stimulation should be increased. In addition, if a rat cyborg is moving too fast, which means the rat cyborg is in underreaction, it may miss the guidance of the training commands (LEFT, RIGHT, and FORWARD), especially at the turns of the maze. In such case, the reward intensity should be decreased to calm the rat cyborg down. In this module, the number of the immobility behavior in each trial (labeled as *N*_*q*_) and the locomotion speed (labeled as *V*_*r*_) of the rat cyborg are always recorded. *V*_*r*_ is calculated by the mean displacement of the body position in 1800 consecutive frames (about 60 seconds).

#### 3.2.4. Adaptive Adjustment

Adaptive adjustment of the reward intensity does not always happen, but it is important. This module would be triggered by the learning states evaluation module to provide the adaptive intensity of reward stimulation based on two rules written by the training expert. The two rules are as follows: too frequent emergence of the immobility behavior (i.e., underreaction) in a trial would trigger the increase of the reward intensity, and too fast a speed (i.e., overreaction) would trigger the decrease of the reward intensity.

Based on the stimulation response of each rat cyborg, we divided the reward stimulation into different reward levels (*L*) [30]. Each rat cyborg had a different safe range of the reward level in [1, *t*], which was figured out by manual test in the stimulation parameters adjustment procedure. Level 1 is the minimum reward level to excite the rat cyborg, and level *t* is the maximum reward level to protect the rat cyborg from being injured by excessively strong reward stimulation. In the safe range, we assume that the higher the level is, the more excited the rat cyborg will be. Note that in the automatic training, the initial reward level of each rat cyborg is not level 1, but the level which has the best reward-seeking response. Each level is adjusted by the parameters of pulse number (*S*) and pulse amplitude (*Z*). Mathematical relationship between the different levels is shown in (1), and the two rules are formalized in (2a) and (2b):

(1) Sk+1=S1+14k,Zk+1=Z1+14k,0≤k≤t−1

(2a) L=L+1,for  Nq≥4

(2b) L=L−1,for  Vr≥80.

## 4. Experiments

### 4.1. System Implementation

**Figure 7:**
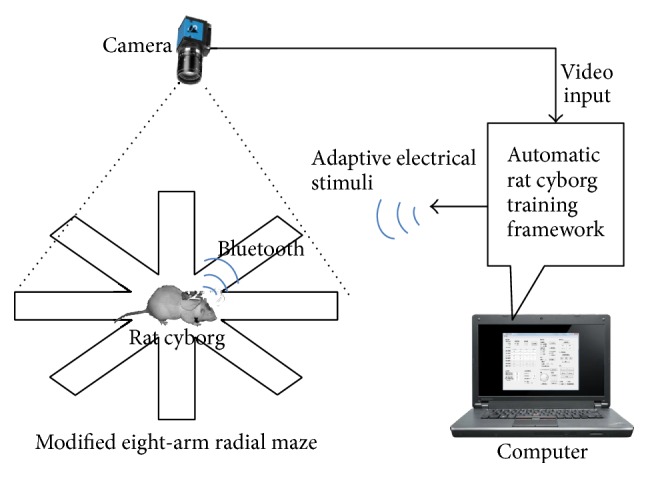
Automatic rat cyborg training system.

**Figure 8:**
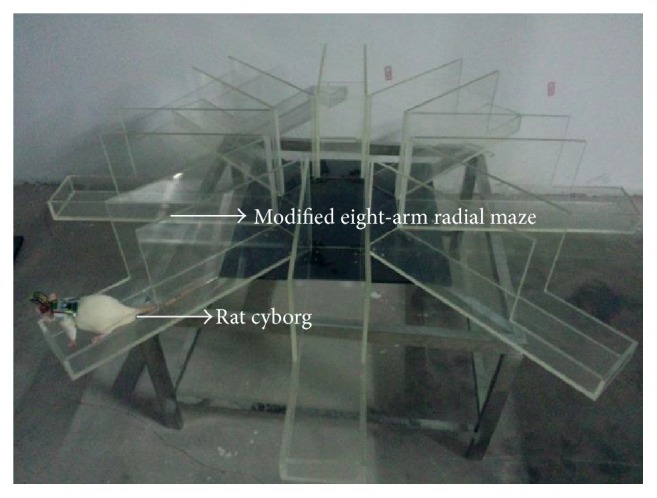
A side view of the modified eight-arm radial maze for automatic training. Two high plexiglass walls (40 cm × 15 cm) have been added to each arm (60 cm × 15 cm) to prevent the rat cyborg from climbing up the arm, and the width of each arm is set small enough to prevent the rat cyborg from turning around. Because the plexiglass walls is shorter than each arm, the rat cyborg can turn around at the tail end.

The automatic rat cyborg training system is shown in Figure 7. A bird-eye camera monitors the whole experimental process and sends real-time video frames to the computer. The automatic training software running on the computer processes the video frames timely and sends adaptive electrical stimuli to the rat cyborg via Bluetooth. The camera we used is The Imaging Source's DFK21BU04. It can capture 30 frames per second, with a resolution of 640 by 480 pixels. The task environment is an eight-arm radial maze which has been modified to facilitate the automatic rat cyborg training (see Figure 8). The automatic training software is implemented in C++ with a user-friendly interface written by Qt.

### 4.2. Steps of Experiments

**Figure 9:**
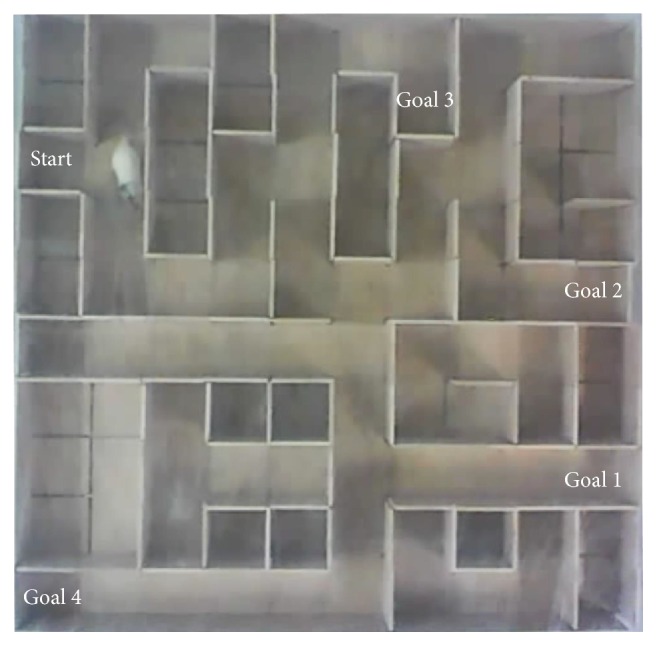
Navigation test maze. This maze is made of wood and comprises 10 × 10 unit squares (15 cm × 15 cm per unit square). The walls of the maze are 15 cm high and the outside walls enclose the entire maze. The starting point and four goals are also presented.

This study was approved by the Ethics Committee of Zhejiang University (Agreement number Zju201402-1-02-034). All applicable institutional and/or national guidelines for the care and use of animals were followed. We carefully conducted our experiments in four steps. In step  1, the best manually trained rat cyborg named F05 was manually navigated by an experimenter along some defined routes in the modified eight-arm radial maze, and the outputs of our automatic training system were checked but not sent to F05. This step ensured the correctness of the electrical stimulus outputs. In step  2, we tested our training framework on F05. It successfully finished T1 and T2 tasks in less than 10 minutes. This step preliminarily ensured the effectiveness of our training system. Steps  1 and 2 were to validate our automatic training system. After the surgery and stimulation parameters adjustment procedures, three adult Sprague Dawley rats (>250 g), namely, T03, DH06, and DH08, proceeded to the automatic navigation training in step  3, which consisted of 4 trials per day with 30 minutes per trial in consecutive days. The sequence of the training was T1_*a*_→T1_*b*_→T2. The completion time of each task and the behavior changes of each rat cyborg were recorded. A demo video of this step is shown in Video S1 of the Supplementary Material available online, and the experimental data of this step are presented in Table S2 of the Supplementary Material available online. Finally, in step  4, rat cyborgs which had successfully completed the training tasks took a manual navigation test in a complex maze (see Figure 9). This step was to exclude the contingencies of training tasks accomplishment and compare the navigation performance of the three rat cyborgs with that of F05. In this step, the rat cyborgs would be manually navigated to four goals one by one from the same starting point (start→goal 1, start→goal 2, start→goal 3, and start→goal 4), and the total time spent was recorded. A demo video of this step is shown in Video S2 of the Supplementary Material available online.

## 5. Results and Discussion

In this section, we first analyze the completion time of each rat cyborg in the training tasks and compare it with that of manual training. Then we measure the total time spent by each rat cyborg in the navigation test and compare it with that of the best manually trained rat cyborg (i.e., F05). In addition, learning curves and behavior changes of each rat cyborg in the automatic training are also presented. These two results which are lacking in the manual training will provide a new insight into the navigation training. Note that we do not make a comparison between manual and automatic training for the same subjects because it is difficult to avoid the interference between these two training procedures. Once a rat cyborg goes through sufficient manual navigation training, it will have the connections established between electrical stimulation and navigation behaviors. Its memory of the previous training will accelerate the training procedure of the automatic training.

### 5.1. Completion Time

Table 2 shows the completion time, which is measured in days and trials, of each training task. Three rat cyborgs all finished the training tasks in no more than 3.5 days, which are less than about 6 days of the manual training and 10 days of Lee et al.'s work [20]. As mentioned in Section 2.2, the first turning task (T1_*a*_) in T1 is decided by the direction rat cyborgs prefer to turn to. T1_*a*_ task for T03 and DH08 is to turn left; for DH06 it is to turn right. From Table 2 we found that rat cyborgs could finish T1_*a*_ task quickly (DH06 took 3 trials and DH08 took 1 trial), but they need more time to finish T1_*b*_ task (DH06 took 8 trials and DH08 took 12 trials). After T1 task, these three rat cyborgs finished mixed turn task (T2) which seemed to be more complex in a short time (DH06 took 2 trials and DH08 took 1 trial). It indicates that the key to the navigation training is to teach the rat cyborgs to turn to the direction they do not prefer, namely, completing T1_*b*_ training task.

### 5.2. Navigation Test

As mentioned in Section 4.2, navigation test is to test the navigation performance of the trained rat cyborgs.  Table 3 shows the total time spent in the navigation test. All of the three rat cyborgs were successfully navigated from the same starting point to the four goals one by one without any mistake. In terms of the total time spent, DH08 even has a better performance compared to F05. This result is an evidence to prove the effectiveness of our automatic training system and demonstrates that our automatic well-trained rat cyborgs can be used in practical navigation applications.

### 5.3. Learning Curves

**Figure 10:**
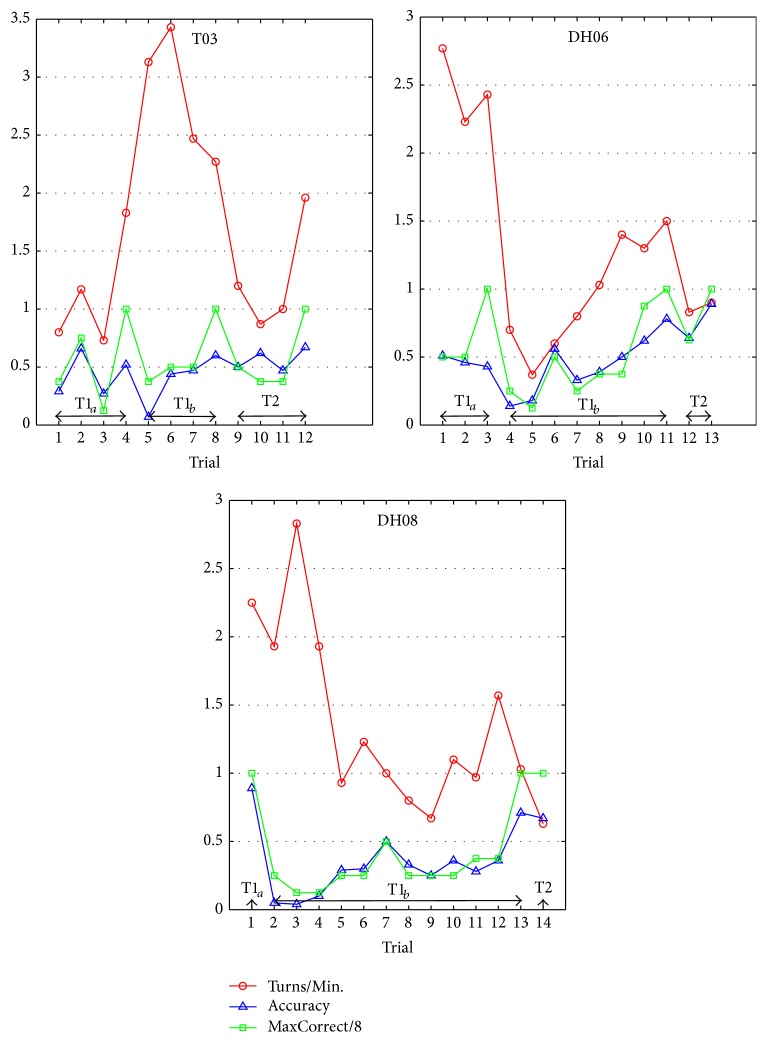
Learning curves of the automatic training.

Figure 10 shows two learning curves (Accuracy and MaxCorrect/8) with the curve of Turns/Min. Turns/Min. means the average counts of arms which have been gone through by the rat cyborg in 1 minute. Accuracy means the ratio of correct turns in a trial. MaxCorrect means the maximum consecutive correct turns in a trial, and a value of 8 means the accomplishment of the current task. The two learning curves (in blue triangle and in green square) can evaluate the learning ability of the rat cyborgs in the training tasks. As we can see from Figure 10, they present an increasing trend in each training task (T1_*a*_, T1_*b*_, and T2) and show a positive correlation with each other (the Pearson's correlation coefficients between the two learning curves of T03, DH06, and DH08 are 0.6404, 0.8005, and 0.9244, resp.). In addition, the curve of Turns/Min. (in red circle) can evaluate, to some extent, the degree of the reward-seeking desire. This curve also shows a positive correlation, although not obvious, with the two learning curves. We speculate that a rat cyborg is more likely to finish the training if it has a strong desire of the reward-seeking. These results are consistent with our manual training experience.

### 5.4. Behavior Changes

**Figure 11:**
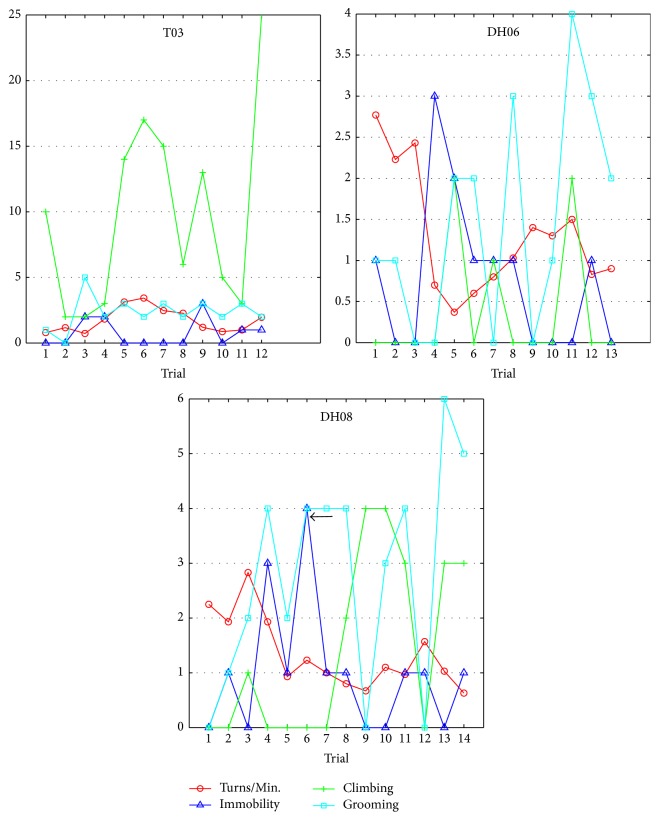
Behavior changes in the automatic training.

Figure 11 shows the changes in numbers of the three behaviors (immobility, climbing, and grooming) in each trial. Numbers of climbing and grooming behaviors were hand counted through observation, and numbers of immobility behavior were counted automatically by our automatic training system. The curve of the immobility behavior (in blue triangle) and the curve of Turns/Min. (in red circle) show a negative correlation with each other (the Pearson's correlation coefficients between these two curves of T03, DH06, and DH08 are −0.3679, −0.4907, and −0.0328, resp.). This is consistent with the fact that a rat cyborg would not stay in immobility if it has a strong desire for the reward. In addition, compared to the other two rat cyborgs, T03 has a large number of the climbing behavior. This phenomenon is probably due to the surgery impact. Most important of all, during the training procedure of DH08, the adaptive adjustment module was triggered to raise the reward level in trial 6, from level 6 (pulse number: 11, pulse amplitude: 5.25) to level 7 (pulse number: 11, pulse amplitude: 5.5) because the number of the immobility behavior reached 4 (pointed by a black arrow). After this reward level adjustment, the number of the immobility behavior was no more than 1 in the succeeding trials. The adjustment of the reward level rarely appeared in our automatic training procedure, the reason being that the initial reward level of each rat cyborg was the one with the best reward-seeking response.

## 6. Conclusions and Future Work

In this study, we build a vision-based automatic training system for rat cyborgs to replace the time-consuming manual training procedure. Training tasks in the navigation training are reset as T1_*a*_, T1_*b*_, and T2, new sensing methods for the tiny rat cyborgs are adopted, and a hierarchical automatic training framework which has a reactive layer and a deliberative layer is proposed. Coadaptation enables continuous, synergistic adaptation between living beings and machines working in changing environments [31–35]. The framework provides a coadaptive learning method for cyborg intelligent systems which combine living beings and machines via BMIs [36–39]. The training rat cyborgs learn to do the behaviors that can obtain the reward, and the machines learn to adjust the electrical stimulation according to the training states and learning states. The experimental results show that our method successfully built the correspondence between the stimuli and the desired behaviors and consumed less training time than that in the previous manual training.

In future work, in order to recognize more behaviors like grooming and climbing, cameras will be mounted beside or under the maze to monitor the rat cyborgs from other perspectives. Our system could be combined with other actuators (such as ultrasonic, vibration generator, and LED photic stimulator) instead of the mild electrical stimuli in SI as directional cues. Furthermore, the parameterized state machine control algorithm could be housed on a wireless backpack stimulator instead of in the computer.

## Supplementary Material

Video S1 is a demo video of the automatic training in T1a, T1b, and T2. Video S2 is a demo video of the automatic training in navigation test. Table S1 presents the parameters of the parameterized state machines. Table S2 presents the experimental data of the automatic training by T03, DH06 and DH08 in T1a, T1b and T2.

## Figures and Tables

**Table 1:** Immobility recognition.

	Immobility	Movement
Immobility	82	2
Movement	18	598

**Table 2:** Completion time of each training task.

Rat cyborg	Completion time							
	T1_*a*_		T1_*b*_		T2		Total	
	Days	Trials	Days	Trials	Days	Trials	Days	Trials
T03	1	4	1	4	1	4	3	12
DH06	0.75	3	2	8	0.5	2	3.25	13
DH08	0.25	1	3	12	0.25	1	3.5	14

**Table 3:** Total time spent in the navigation test.

	Manual	Automatic		
	F05	T03	DH06	DH08
Total time spent (s)	232	245	330	225

F05 is the best manually trained rat cyborg.
