# Supplementary material for: Automatic Training of Rat Cyborgs for Navigation
Source: Comput Intell Neurosci. 2016 Jun 29;2016:6459251. doi: 10.1155/2016/6459251 (PMC4942600; doi:10.1155/2016/6459251)
Supplement: Supplementary file 1 — Video S1 is a demo video of the automatic training in T1a, T1b, and T2. Video S2 is a demo video of the automatic training in navigation test. Table S1 presents the parameters of the parameterized state machines. Table S2 presents the experimental data of the automatic training by T03, DH06 and DH08 in T1a, T1b and T2. [file 6459251.f1.zip › Supplementary Material/Ethical Approval Form in Chinese and English.pdf]

2. The authorization file of 2014.

## 浙江大学医学部实验动物

## 伦理委员会项目审批件

| | | | |
|---|---|---|---|
| 项目名称 | 脑机融合感知和认知的计算理论与方法 | | |
| 申请部门 | 浙江大学计算机学院 CCNT 实验室 | 项目负责人： 吴朝晖 | |
| | | 参加研究人员： 潘纲，王跃明，许科帝等 | |
| 报送材料 | 科研项目伦理审查申请表☐ √ 课题申报书☐ 研究方案和项目摘要☐ | | |
| 审查 | 研究者资格：符合要求☑ 不符合要求☐ | 项目来源：国家自然科学基金委 | |
| | 试验方案：适当☑ 不适当☐ | | |

| 伦理委员会委员签名 | 委员姓名 | 签名 | 委员姓名 | 签名 | 委员姓名 | 签名 |
|---|---|---|---|---|---|---|
| | 沈华浩 | 沈华浩 | 谢强敏 | 谢强敏 | 周天华 | [illegible] |
| | 柯越海 | 柯越海 | 何俏军 | 何俏军 | 施卫星 | 施卫星 |
| | 陆 源 | 陆源 | 裴增杨 | | 马 骏 | [illegible] |

| | |
|---|---|
| 结果 | 参会 8 人，投票 8 人；<br>同意 8 票，作必要修正后同意____票，修改后再次会议讨论____票，不同意____票。<br>* 是否启动紧急程序：是☐ 否☐ |

| 结论 | 同意 | 同意 稍作修改 | 修改后再次会议讨论 | 不同意 |
|---|---|---|---|---|
| | √ | | | |

批准号：**Zju201402-1-02-034**

审评意见：

同意

主任委员（签名）：[illegible]

浙江大学医学部伦理委员会（盖章）：

2014 年 3 月 3 日

* 注：根据《浙江大学医学部实验动物伦理审查委员会章程》第二十二条规定，遇到紧急（时间紧）项目，经主任委员批准启动紧急审查程序：常务副主任委员和 3 名及以上委员审查，形成审查决议，由常务主任委员签发。

3. The English translation of the authorization file of 2014.

# Zhejiang University Ethics Committee

# Ethical Approval Form

<table>
<tr><td>Project Title</td><td colspan="6">Computational Theory and Methods for Brain-machine-integrated Perception and Cognition</td></tr>
<tr><td rowspan="2">Department</td><td colspan="2" rowspan="2">CCNT Lab, College of Computer Science</td><td colspan="4">Project Investigator：Zhaohui Wu</td></tr>
<tr><td colspan="4">Other Researchers：Gang Pan, Yueming Wang, Kedi Xu, et al.</td></tr>
<tr><td>Submitted Documents</td><td colspan="6">Application Form √ Project Proposal☐ Project Plan☐</td></tr>
<tr><td rowspan="2">Review</td><td colspan="4">Qualification: Qualified √ Unqualified☐</td><td colspan="2" rowspan="2">Financial Source：<br>National 973 Program</td></tr>
<tr><td colspan="4">Experiment Plan: Appropriate √ Inappropriate☐</td></tr>
<tr><td rowspan="4">Committee Member & Signature</td><td>Name</td><td>Signature</td><td>Name</td><td>Signature</td><td>Name</td><td>Signature</td></tr>
<tr><td>Huahao Shen</td><td></td><td>Qiangmin Xie</td><td></td><td>Tianhua Zhou</td><td></td></tr>
<tr><td>Yuehai Ke</td><td></td><td>Qiaojun He</td><td></td><td>Weixing Shi</td><td></td></tr>
<tr><td>Yuan Lu</td><td></td><td>Zengyang Pei</td><td></td><td>Jun Ma</td><td></td></tr>
<tr><td>Voting Result</td><td colspan="6">Members participated__8__, members voted__8___.<br>Approval__8___, approval with revision______, resubmission_____, disapproval_____.<br>*Emergency program: Yes□ No□</td></tr>
<tr><td rowspan="2">Decision</td><td>Approval</td><td colspan="2">Approval with revision</td><td colspan="2">Resubmission</td><td>Disapproval</td></tr>
<tr><td>✓</td><td colspan="2"></td><td colspan="2"></td><td></td></tr>
<tr><td colspan="7">Certification Number: Zju201402-1-02-034</td></tr>
<tr><td colspan="7">Comments:<br><br>Chair (Signature): Huahao Shen<br>Zhejiang University Ethics Committee (seal):<br>Date: March 3rd, 2014</td></tr>
</table>

*According to the item 22 of the Ordinances Of Zhejiang University Ethics Committee, in case of emergency, the Ethical Approval Form is allowed to be censored by the executive vice chair and at least 3 committee members, then signed and issued by the chair.

4. A brief introduction to the members of the ethics committee.

| | | | |
|---|---|---|---|
| Huahao Shen | Chair | Professor | Zhejiang University School of Medicine |
| Qiangmin Xie | Executive Vice Chair | Professor | Zhejiang University Laboratory Animal Center |
| Tianhua Zhou | Member | Professor | Zhejiang University School of Medicine |
| Yuehai Ke | Member | Professor | Zhejiang University School of Medicine |
| Qiaojun He | Member | Professor | Zhejiang University Good Laboratory Practice of Drug (GLP) |
| Weixing Shi | Member | Professor | Zhejiang University School of Medicine, Medical Ethics and Health Laws |
| Yuan Lu | Member | Animal Research Associate | Zhejiang University School of Medicine |
| Zengyang Pei | Member | Veterinarian | Zhejiang University Veterinary Teaching Hospital |
| Jun Ma | Member | Lawyer | Grandall Law Firm |
